# Molecular Detection and Identification of Spotted Fever Group Rickettsiae in Ticks Collected from the West Bank, Palestinian Territories

**DOI:** 10.1371/journal.pntd.0004348

**Published:** 2016-01-15

**Authors:** Suheir Ereqat, Abedelmajeed Nasereddin, Amer Al-Jawabreh, Kifaya Azmi, Shimon Harrus, Kosta Mumcuoglu, Dimtry Apanaskevich, Ziad Abdeen

**Affiliations:** 1 Biochemistry and Molecular Biology Department, Faculty of Medicine, Al-Quds University, Abu Deis, the West Bank, Palestinian territories; 2 Al-Quds Public Health Society, Jerusalem, Palestinian territories; 3 Arab American University, Jenin, Palestine; 4 Koret School of Veterinary Medicine, The Hebrew University of Jerusalem, Rehovot, Israel; 5 Department of Microbiology and Molecular Genetics, The Hebrew University - The Kuvin Center for the Study of Infectious and Tropical Diseases, Hadassah Medical School, Jerusalem, Israel; 6 United States National Tick Collection, Institute for Coastal Plain Science, Georgia Southern University, Statesboro, Georgia, United States of America; Faculté de Médecine,Aix-Marseille Université, FRANCE

## Abstract

**Background:**

Tick-borne rickettsioses are caused by obligate intracellular bacteria belonging to the spotted fever group (SFG) rickettsiae. Although Spotted Fever is prevalent in the Middle East, no reports for the presence of tick-borne pathogens are available or any studies on the epidemiology of this disease in the West Bank. We aimed to identify the circulating hard tick vectors and genetically characterize SFG *Rickettsia* species in ixodid ticks from the West Bank-Palestinian territories.

**Methodology/Principal Findings:**

A total of 1,123 ixodid ticks belonging to eight species (*Haemaphysalis parva*, *Haemaphysalis adleri*, *Rhipicephalus turanicus*, *Rhipicephalus sanguineus*, *Rhipicephalus bursa*, *Hyalomma dromedarii*, *Hyalomma aegyptium* and *Hyalomma impeltatum*) were collected from goats, sheep, camels, dogs, a wolf, a horse and a tortoise in different localities throughout the West Bank during the period of January-April, 2014. A total of 867 ticks were screened for the presence of rickettsiae by PCR targeting a partial sequence of the *ompA* gene followed by sequence analysis. Two additional genes, *17 kDa* and *16SrRNA* were also targeted for further characterization of the detected *Rickettsia* species. Rickettsial DNA was detected in 148 out of the 867 (17%) tested ticks. The infection rates in *Rh*. *turanicus*, *Rh*. *sanguineus*, *H*. *adleri*, *H*. *parva*, *H*. *dromedarii*, and *H*. *impeltatum* ticks were 41.7, 11.6, 16.7, 16.2, 11.8 and 20%, respectively. None of the ticks, belonging to the species *Rh*. *bursa* and *H*. *aegyptium*, were infected. Four SFG rickettsiae were identified: *Rickettsia massiliae*, *Rickettsia africae*, *Candidatus* Rickettsia barbariae and *Candidatus* Rickettsia goldwasserii.

**Significance:**

The results of this study demonstrate the geographic distribution of SFG rickettsiae and clearly indicate the presence of at least four of them in collected ticks. Palestinian clinicians should be aware of emerging tick-borne diseases in the West Bank, particularly infections due to *R*. *massiliae* and *R*. *africae*.

## Author Summary

Tick borne rickettsial diseases may have similar clinical characteristics, yet epidemiologically and etiologically different diseases. To date, no studies have been conducted to detect potential tick vectors of rickettsiae in the West Bank. Therefore, we aimed to identify tick species and to determine the presence of *Rickettsia* pathogens in naturally infected ixodid ticks. The overall prevalence of SFG rickettsiae detected in ixodid ticks in nine Palestinian districts was 17%. Our results document for the first time the finding of two important human pathogens carried by ixodid ticks in the West Bank: *R*. *massiliae* and *R*. *africae*, the agent of African tick bite fever. Genetically, the detected *Rickettsia* spp. clustered into 3 different groups: *R*. *massiliae*, *C*. R. barbariae and *C*. R. goldwasserii. Most of *Rickettsia*-infected ticks were collected from dogs, camels and sheep, increasing the risk of transmitting rickettsial infections to animal owners, shepherds and farmers. These findings highlight the importance of hard ticks and their potential hazard for human health in the West Bank.

## Introduction

Tick-borne spotted fever group (SFG) rickettsioses are caused by obligate intracellular Gram-negative bacteria belonging to the genus *Rickettsia* [1]. Feeding ticks can transmit these microorganisms to humans and animals. Various vertebrates are suspected to serve as reservoirs for *Rickettsia* species; however, some are susceptible to rickettsial infections and may develop rickettsemia following tick bite [2]. The human disease may present as a fever with clinical symptoms including headache, rash, and occasional eschar formation at the site of the tick bites [3].

Mediterranean spotted fever (MSF), caused by *Rickettsia conorii*, is transmitted by the brown dog tick, *Rhipicephalus sanguineus*, which is well adapted to urban environments. Previous studies in Israel have documented the presence of two spotted -fever group (SFG) rickettsiae: the tick-borne rickettsia, *Rickettsia conorii israelensis* and the flea-borne rickettsia, *Rickettsia felis* [4], [5]. *Rickettsia conorii israelensis* has been described in Tunisia, Libya, Sardinia-Italy, and Portugal [6]. Furthermore, a number of other SFG pathogenic rickettsiae including *Rickettsia africae*, *Rickettsia massiliae* and *Rickettsia sibirica mongolitimonae* have been detected in ticks from Israel in addition to some rickettsial species such as *Candidatus* Rickettsia barbariae and *Candidatus* Rickettsia goldwasserii which were not associated with diseases, to date [7], [8], [9].

The number of newly described SFG rickettsiae has increased in recent decades [4]. Sequence analysis of PCR-amplified fragments targeting genes encoding the citrate synthase (*gltA*) [10], *Rickettsia*-specific outer membrane protein (*ompA*) [11], the 17kDa lipoprotein precursor antigen gene (*17 kDa*) [12], and the ribosomal *16S rRNA* gene [13] has become a reliable method for the identification of *Rickettsia* species. Molecular typing of infectious agents is important for better understanding of ecological niches and identifying circulating strains and their virulence. Although various *Rickettsia* species are found in ticks from Israel; to date, no entomological survey has been carried out in the West Bank, and no clinical data or reports for the presence of tick-borne pathogens are available. Thus, this study aimed at identification of the circulating hard tick vectors and *Rickettsia* species in naturally infected ixodid ticks collected from the West Bank, using PCR and sequence analysis with special focus on their potential threat for humans and animals.

## Materials and Methods

### Study area and ticks

**Fig 1:**
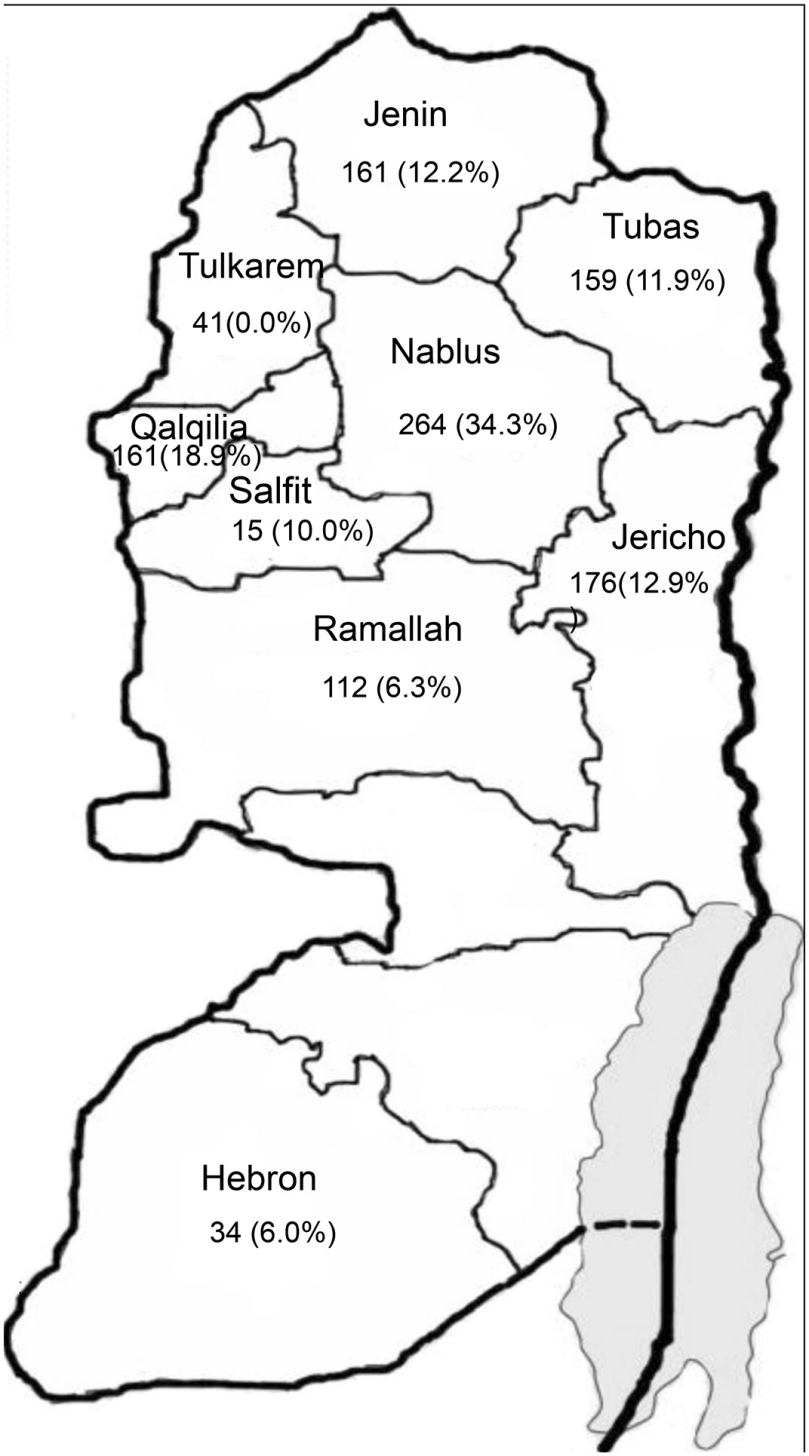
Distribution of ticks in the West Bank-Palestinian territories from which rickettsial DNA was detected. Percentage indicates the overall infection rate of tick populations in each district.

To identify the circulating hard ticks in the West Bank and to evaluate the presence of rickettsial infection in these ticks, one to ten hard ticks per animal host, for a total of 1,123, were collected during January to April, 2014 from dogs, camels, sheep, a horse, a wolf, and a tortoise residing in nine districts in the West Bank. The districts are located in three zones in the central, northern and southern regions of the country (Fig 1). All ticks were gently removed from their hosting animals by forceps or hand, and individually placed into small, labeled plastic tubes containing 70% ethanol for morphological identification. The ticks were identified using standard taxonomic keys [14], [15], [16], [17] and stored at −20°C until DNA extraction.

### DNA extraction

A maximum of five ticks of different tick species per hosting animal were randomly selected for DNA extraction. Genomic DNA was individually extracted from a total of 867 tick samples. Prior DNA extraction, individual ticks were washed with phosphate-buffered saline (PBS), air dried for 10 min on tissue paper and separately sliced into small pieces by a sterile scalpel blade then manually homogenized with a sterile micro pestle, resuspended in 200 μl of lysis buffer and 20 μl of proteinase K. After overnight incubation at 56°C with a continuous gentle shaking, the DNA was extracted using the QIAamp DNA tissue extraction kit (Qiagen, Hilden, Germany) following the manufacturer's protocol. Purified DNA was stored at 4°C until use. Three μl of template DNA (approximately 100–200 ng per tick) were used for each PCR.

### Molecular detection of *Rickettsia* species

Screening for the presence of rickettsial DNA was carried out by conventional PCR targeting a 250-bp fragment of the *ompA* gene using 107F and Rm299 primers as described previously [7] with the following modification: final volume of 25μl using PCR-Ready Supreme mix (Syntezza Bioscience, Jerusalem) including primers at 1μM final concentration. Positive samples were further characterized targeting a 426-bp portion of the *16SrRNA* and a 265-bp portion of the *17kDa* protein gene as previously described [18], [19]. For *Rickettsia* species identification, strong positive samples were sent for sequencing. DNA extract of the first *ompA* positive sample, identified as *R*. *massiliae*, by direct sequencing, was used as a positive control and ultra pure water were used as a negative control in each amplification reaction.

### Phylogenetic analysis

DNA sequences of the positive PCR products were assembled using Bioedit software, used in a BLAST search (ncbi.nlm.nih.gov/blast) and aligned with sequences of other rickettsial species registered in the GenBank. To infer relationships between the obtained amplicons and other reference sequences published in GenBank, a phylogenetic tree was constructed using MEGA6 program.

### Statistical tests

Statistical analysis was done using the SPSS program v20. Two –tailed t- test and Pearson’s correlation were performed. P-value <0.05 was considered statistically significant.

### Ethics statement

The animal population was residing in different farms throughout the West Bank. Prior to ticks sampling, the animal owners were verbally informed about the goals of the project and the sampling protocol. All owners gave their verbal informed consent to collect ticks from their animals. The study was approved by the ethics committee at the Faculty of Medicine in Al-Quds University-Palestine (EC number: ZA/196/013).

## Results

### Sampling and identification of ticks

A total of 1,123 hard ticks were collected from 320 animals (234 dogs, 68 sheep, 10 camels, 5 goats, one horse, one wolf and one Mediterranean spur-thighed tortoise). From each infested animal one to ten ticks were collected. Of them, 547 were male ticks (48.7%), 511 females (45.5%) and 65 nymphs (5.8%). All tick samples were identified to the species level as follows: *Rhipicephalus sanguineus* (n = 694), *Rhipicephalus turanicus* (n = 191), *Rhipicephalus bursa* (n = 16), *Rhipicephalus* spp. (n = 21), *Haemaphysalis parva* (n = 100), *Haemaphysalis adleri* (n = 20), *Hyalomma dromedarii* (n = 68), *Hyalomma impeltatum* (n = 5), *Hyalomma aegyptium* (n = 4), and *Hyalomma* spp. (n = 4) (Table 1).

**Table 1:** Morphological identification of the collected ticks and their associated host animal species.

Animal species	Tick species (number)	Total
Dog	*Rh*. *sanguineus* (634), *Rh*. *turanicus* (53), *Rh*. *bursa* (1), *Rh*. spp.(15), *H*. *parva* (93), *H*. *adleri* (19)	518
Sheep	*Rh*. *turanicus* (138), *Rh*. *sanguineus* (41), *Rh*. *bursa* (14), *Rh*. spp.(6), *H*. *parva* (4), *H*. *adleri* (1)	204
Camel	*H*. *dromedarii* (68), *H*. *impeltatum* (5), *Hyalomma* spp. (4).	77
Goat	*Rh*. *sanguineus* (16), *Rh*. *bursa* (1), *H*. *parva* (3)	20
Horse	*Rh*. *sanguineus* (2)	2
Tortoise	*H*. *aegyptium* (4)	4
Dead wolf	*Rh*. *sanguineus* (1)	1
Total	*Rh*. *sanguineus* (694), *Rh*. *turanicus* (191), *Rh*.spp.(21), *Rh*. *bursa* (16), *H*. *parva* (100), *H*. *adleri* (20), *H*. *dromedarii* (68), *H*. *impeltatum* (5), *Hyalomma* spp. (4), *H*. *aegyptium* (4),	1123

### Screening of ticks for rickettsial DNA

A set of 867 ticks comprising eight different species were screened for rickettsial DNA targeting *Rickettsia*–specific *ompA* (Table 2). A sample was considered positive when PCR yielded a fragment with the expected length (250 bp) of the *ompA* rickettsial gene. The overall prevalence of rickettsiae infection was 17% (148/867) in all ticks (Table 2). The detection of rickettsial DNA was significantly higher in female ticks (20.4%) than in male (15.7%) and nymph ticks (4.8%, p<0.01). The overall prevalence of rickettsial DNA was markedly higher in ticks collected from Nablus (34.3%; 66/192) compared to other districts (p<0.01) (Fig 1).

**Table 2:** The overall prevalence of Rickettsial DNA and molecular identification of SFG *Rickettsia* in tick species picked from different hosts from different districts in the West Bank-Palestinian territories.

Ectoparasites	#PCR Positives (%)	Animal host	Collection site	*Ricketssia* sp. detected
*Rh*. *turanicus*	60(41.7)	Dog, Sheep	Jericho, Nablus, Qalqilia, Tubas, Ramallah	*R*. *massiliae*, *C*. R.barbariae, *C*.R. goldwasserii
*Rh*. *sanguineus*	61(11.6)	Dog, Sheep, Goat, Horse, wolf	Jericho, Nablus, Jenin,Qalqilia, Tubas, Ramallah	*R*. *massiliae*, *C*. R.barbariae, *C*.R. goldwasserii
*Rh*. spp.	3(15.8)	Dog, Sheep	Nablus	*Rickettsia spp*.
*H*. *adleri*	3(16.7)	Dog, Sheep	Jenin,Qalqilia,	*R*. *massiliae*, *C*.R. goldwasserii
*H*. *parva*	11(16.2)	Dog, Sheep	Nablus, Jenin,Qalqilia, Ramallah	*R*. *massiliae*, *C*.R. goldwasserii
*H*. *dromedarii*	8(11.8)	Camel	Jericho	*R*. *africae*, *C*. R. barbariae
*H*. *impeltatum*	1(20)	Camel	Jericho	*R*. *africae*
*Hyalomma* spp.	1(25)	Camel	Jericho	*Rickettsia spp*.
Total ticks	148 (17)			

The infection rates in *Rh*. *turanicus*, *Rh*. *sanguineus*, *Haemaphysalis adleri*, *H*. *parva*, *Hyalomma dromedarii* and *H*. *impeltatum* ticks were 41.7, 11.6, 16.7, 16.2, 11.8 and 20% respectively. None of the ticks belonging to *Rh*. *bursa* (0/13) and *H*. *aegyptium* (0/4), taken from sheep and one tortoise, respectively, were infected (Table 2).

### Identification of the *Rickesttia* species

Among the positive samples (n = 148), identification of rickettsial DNA based on sequencing of the *ompA* amplicons were successfully obtained from 63 (42.6%) samples which showed strong bands on agarose gel. These samples were subsequently subjected to two additional amplification reactions targeting the *16SrRNA* and *17kDa* genes of *Rickettsia* species. Successful sequences were only obtained from (35/63) and (36/63) by *16SrRNA* and *17kDa* PCR, respectively.

**Fig 2:**
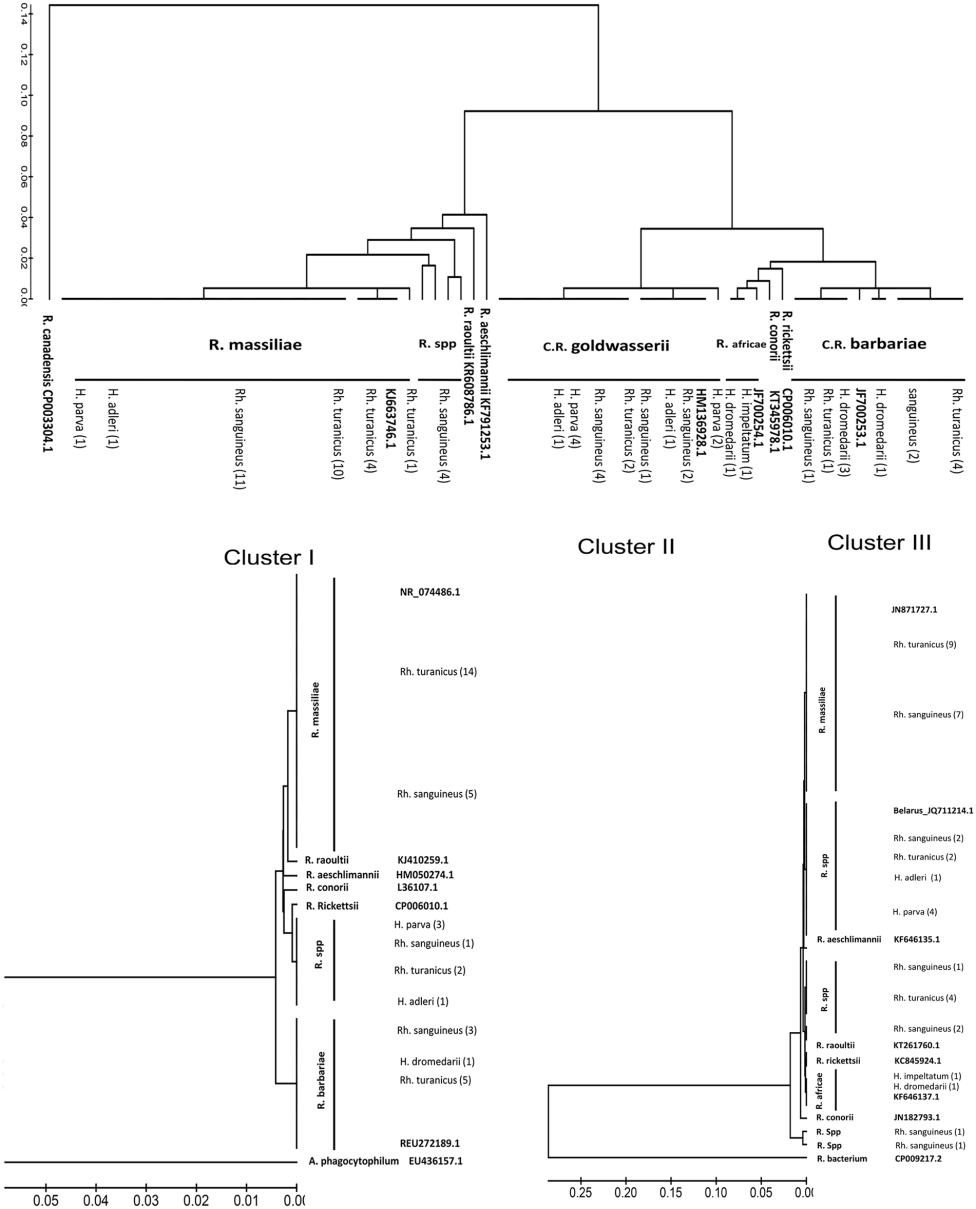
Phylogenetic analyses of *Rickettsia* species detected in the West Bank-Palestinian territories based on partial sequences of *ompA*, *16SrRNA* and *17kDa* genes. MEGA 6.0 program was used for constructing the Phylogenetic trees. DNA sequences were aligned using ClustalW (1.6) program, the trees were built using UPGMA statistical method with 1000 bootstrap using maximum composite likelihood model, comparing panels: (A) 217 bp *OmpA* (B) 371 bp *16SrRNA* (C) 248 bp *17kDa* DNA *Rickettsia* sequences from ticks collected in this study to the respective *Rickettsia* reference sequences deposited in the NCBI GenBank. *R*. *canadensis* (CP003304.1), *Anaplasma phagocytophilum* (EU436157.1) and *R*. *bacterium* (CP009217.2) was used as out-groups. Species of ticks and the number of identical sequences from the same tick species was indicated. All sequences identified in this study were derived from the West Bank.

BLAST analysis of the positive *ompA* sequences revealed 4 different rickettsial species. Twenty eight ticks were tested positive for *R*. *massiliae*-DNA including 15 *Rh*. *turanicus*, 11 *Rh*. *sanguineus*, one *Haemaphysalis parva* and one *H*. *adleri*, all obtained from dogs and sheep. *C*. R. barbariae-DNA was found in 12 ticks: 5 *Rh*. *turanicus*, 3 *Rh*. *sanguineus* and 4 *H*. *dromedarii*. The DNA of *C*. R. goldwasesrii- was detected in 17 ticks: 2 *Rh*. *turanicus*, 7 *Rh*. *sanguineus*, 6 *H*. *parva* and 2 *H*. *adleri*. Four ticks were positive for *Rickettsia* species found in four *Rh*. *sanguineus* ticks, Furthermore, *R*. *africae*-DNA was detected in two ticks, *H*. *impeltatum* and *H*. *dromedarii* obtained from two different camels in Jericho (Fig 2A). There were no cases in which multiple rickettsiae species were detected in the same infected tick.

### Phylogenetic analysis

Phylogenetic analysis based on *ompA* sequences revealed three main clusters: *R*. *massiliae*, *C*. R. barbariae *and C*. R. goldwasserii. Cluster I, representing the *R*. *massiliae* group (n = 28). In this cluster, the nucleotide *ompA* sequences of *R*. *massiliae* identified in this study were identical to each other and to the respective *R*. *massiliae* reference sequence (accession no. KJ663746.1) deposited in the NCBI GenBank (Fig 2A). The same samples also showed one cluster and had 100% similarity to the *R*. *massiliae* reference strains (NR074486.1 and JN871727.1) based on the *16sRNA* and *17kDa* analysis, respectively (Fig 2B and 2C). The DNA sequence of *Rickettsia canadensis* (CP003304.1) that do not belong to the SFG [20], was used as an out group in the analysis of *ompA* gene while the sequences of *Anaplasma phagocytophilum* (EU 436157.1) and Rickettsiaceae bacterium (CP009217.2) were used as out groups in the analyses of the *16SrRNA and 17kDa* genes, respectively. On the basis of the o*mpA* sequences, DNA sequences of four amplicons (1.4G, 1.4H, 14.8 A, 14.8 B) had several nucleotide differences and showed 96–98% sequence identity to the *ompA* sequences of *R*. *massiliae* identified in this study and to the reference strain of *R*. *massiliae* (KJ663746.1). These samples showed 92% and 89% sequence identity to the *ompA* sequences of the reference strain sequences of *R*. *aeschlimannii* (KF791253.1) and *R*. *raoultii* (KR608786.1), respectively. The four samples were further characterized by *17kDa* and *16S rRNA* genes, the partial *17kDa* gene sequence of these samples revealed 94% and 93% similarity to the reference strain sequences of *R*. *raoultii* (KT261760.1) and *R*. *conorii* (JN182793.1), respectively. Two of them (14.84A and 14.8B) formed a separate branch with in this group complicating the further confirmation of this *Rickettsia* spp. (Fig 2C). However, none of these samples (n = 4) were successfully sequenced based on *16SrRNA* gene.

Cluster II represents the *C*. R. goldwasserii group. The *ompA* sequences had 100% similarity to the *C*. R. goldwasserii reference sequence (HM136928.1) whilst the *17kDa* sequences of the same samples had 100% similarity to the corresponding sequence of an incompletely described *Rickettsia* sp. *Belarus* (JQ711214.1) (Fig 2C). The *16SrRNA* sequences showed 99% similarity to that of *R*. *rickettsii* (NR103923). Cluster III represents the *C*. R. barbariae group; the *ompA* and *16SrRNA* sequences had 100% similarity to the *Candidatus* Rickettsia barbariae reference sequences (JF700253.1 and EU272189.1), respectively (Fig 2A and 2B). When *17kDa* sequences were obtained from the same samples, they showed 99% sequence similarity to the homologous fragments of *R*. *raoultii* (KT261760.1) and to the other unidentified *Rickettsia* sp. (KM386654.1) as revealed by BLAST analysis.

A sub-cluster of two *ompA* sequences was identified as *R*. *africae* and showed 100% sequence similarity with a homologous fragment of *R*. *africae* (JF700254) detected in *Hyalomma detritum* from the Golan Heights [9]. The *17kDa* sequences of these two samples had also 100% nucleotide identity to that of *R*. *africae* (KF646137.1) but they were not detected by the *16S rRNA* PCR.

## Discussion

The overall prevalence of SFG rickettsiae detected in ixodid ticks in nine Palestinian districts was 17%. Ticks detected in this study belonged to three genera (*Rhipicephalus*, *Haemaphysalis* and *Hyalomma*) and collected from different host animals. The findings of this study highlight the importance of hard ticks for human health in the West Bank. *Rhipicephalus sanguineus* was the most prevalent tick species found in this study. It parasitized a wide range of mammals including dogs, sheep, goats and horses; however the main vector for SFG rickettsiae detected in this study was *Rh*. *turanicus*. Our results document the detection of two important human pathogens in ticks from the West Bank, *R*. *massiliae* and *R*. *africae*. *R*. *massiliae* was the most prevalent rickettsial species detected in this study. This pathogen was first isolated from *Rh*. *sanguineus* in Marseille in 1992 [21], and since then it has been detected in ticks within the genus *Rhipicephalus* in Greece, Spain, Portugal, Switzerland, Sardinia (Italy), Morocco and Israel [22], [23], [4], [24], [25], [7]. The first isolation of *R*. *massiliae* was reported from a human patient in Sicily in 1985 and identified in 2005 [26]. The clinical presentation of *R*. *massiliae* infection has been previously described [23],[27],[28]. Common clinical signs include fever, night sweats, headache, maculopapular rash and necrotic eschar at the tick bite site. In agreement with other studies, all ticks infected with this pathogen in this study belonged to the genus *Rhipicephalus* except for two which belonged to the genus *Haemaphysalis* obtained from the same dog in Jenin district. However, detection of *R*. *massiliae* in *Haemaphysalis punctata* ticks was previously reported in southeast England [29].

In this study, most of *R*. *massiliae-* infected ticks were removed from dogs (77%) and to a lesser extent from sheep (23%), increasing the risk of transmitting rickettsial infections to the animal owners, i. e. dog owners, shepherds and farmers. The phylogenetic tree based on partial DNA sequences of *ompA*, *16SrRNA and 17 kDa* showed higher genetic variability among the *R*. *massiliae* strains using *ompA* gene than *16SrRNA and 17 kDa* loci. The second SFG pathogenic *Rickettsia* found in this study was *R*. *africae*, the agent of African tick bite fever. The detection of *R*. *africae*, in *Hyalomma* spp. collected from camels in the West Bank confirms the results of a previous report associating *R*. *africae* with *Hyalomma* ticks in Egypt and Israel [30], [8], [9]. Livestock movements and migratory birds may play a role in the geographic spread of *R*. *africae* [31]. We observed a strong geographic correlation between the overall prevalence of rickettsial DNA in ticks collected from Nablus (Northern district) compared to the prevalence of infected ticks collected from other districts in the north, Ramallah (centre) and Hebron (south). Future studies with high representative number of ticks are required to address the comparative importance of geographic distribution on the infection rate of these ticks in the West Bank.

*Candidatus*. R. goldwasserii was also detected in *Rhipicephalus* ticks collected from dogs in the northern region of the West Bank. This *Rickettsia* was first detected in two *Haemaphysalis* ticks (*H*. *adleri* and *H*. *parva*) from golden jackals in Israel. Phylogenetic analysis based on concatenated four gene fragments (*gltA-ompA-sca4-ompB*) indicated that the nucleotide sequences of these SFG rickettsiae belonged to a novel phylogenetic lineage related to *C*. R. siciliensis detected in *Rh*. *turanicus* ticks. [32]. In the present study, the identification of *C*. R. goldwasserii in *Rh*. *sanguineus* and *Rh*. *turanicus*, in addition to the already known *Haemaphysalis* spp, expands the current knowledge concerning tick species that host *C*. R. goldwasserii in our region. Based on *ompA* phylogeny, high genetic homology was observed among the *C*. R. goldwasserii group identified in this study. However, these samples were found to be 100% similar to the corresponding sequence of a not well characterized *Rickettsia* sp. *Belarus* and 99% similar to that of *R*. *rickettsii* based on *17kDa* and *16srRNA* respectively. Thus, for a more accurate classification of this uncultivated SFG *Rickettsia*, further testing and phylogenetic analysis with additional genes is needed since no sequences of these two latter genes of *C*. R. goldwasserii were available in the GenBank.

This is the first study to report the presence of *C*. R. barbariae in 9.6% of *Hyalomma* ticks in the West Bank. The presence of *C*. R. barbariae has been previously reported in several *Rhipicephalus* spp. in Portugal, Italy, France, Cyprus and later in *Rhipicephalus* ticks flagged from the vegetation in Israel [24],[33],[2], [9]. However, no *ompA* sequence differences were observed in *C*. R. barbariae DNA detected in *Hyalomma* or *Rhipicephalus* ticks collected from camels, dogs and sheep residing in different localities throughout the West Bank. In conclusion, the findings presented in this study provide evidence for the presence of *R*. *massiliae* and *R*. *africae* in different ixodid ticks collected from various regions in the West Bank. In addition to *Rhipicephalus* species, members of the genus *Hyalomma* and *Haemaphysalis* may also play an important role in the epidemiology of SFG *Rickettsia* spp. Clinicians in the West Bank and neighboring countries should consider a range of SFG diseases in the differential diagnoses of patients present with fever of unknown origin and clinical signs compatible with rickettsioses.

## References

[1] Azad AF, Beard CB (1998) Rickettsial pathogens and their arthropod vectors. Emerg Infect Dis 4: 179–186. 9621188 10.3201/eid0402.980205 PMC2640117

[2] Chochlakis D, Ioannou I, Sandalakis V, Dimitriou T, Kassinis N, et al (2012) Spotted fever group Rickettsiae in ticks in Cyprus. Microb Ecol 63: 314–323. 10.1007/s00248-011-9926-4 21833539

[3] Brouqui P, Bacellar F, Baranton G, Birtles RJ, Bjoersdorff A, et al (2004) Guidelines for the diagnosis of tick-borne bacterial diseases in Europe. Clin Microbiol Infect 10: 1108–1132. 15606643 10.1111/j.1469-0691.2004.01019.x

[4] Parola P, Paddock CD, Raoult D (2005) Tick-borne rickettsioses around the world: emerging diseases challenging old concepts. Clin Microbiol Rev 18: 719–756. 16223955 10.1128/CMR.18.4.719-756.2005 PMC1265907

[5] Bauer O, Baneth G, Eshkol T, Shaw SE, Harrus S (2006) Polygenic detection of Rickettsia felis in cat fleas (Ctenocephalides felis) from Israel. Am J Trop Med Hyg 74: 444–448. 16525105

[6] Parola P, Paddock CD, Socolovschi C, Labruna MB, Mediannikov O, et al (2013) Update on tick-borne rickettsioses around the world: a geographic approach. Clin Microbiol Rev 26: 657–702. 10.1128/CMR.00032-13 24092850 PMC3811236

[7] Harrus S, Perlman-Avrahami A, Mumcuoglu KY, Morick D, Baneth G (2011) Molecular detection of Rickettsia massiliae, Rickettsia sibirica mongolitimonae and Rickettsia conorii israelensis in ticks from Israel. Clin Microbiol Infect 17: 176–180. 10.1111/j.1469-0691.2010.03224.x 20331680

[8] Kleinerman G, Baneth G, Mumcuoglu KY, van Straten M, Berlin D, et al (2013) Molecular detection of Rickettsia africae, Rickettsia aeschlimannii, and Rickettsia sibirica mongolitimonae in camels and Hyalomma spp. ticks from Israel. Vector Borne Zoonotic Dis 13: 851–856. 10.1089/vbz.2013.1330 24107206

[9] Waner T, Keysary A, Eremeeva ME, Din AB, Mumcuoglu KY, et al (2014) Rickettsia africae and Candidatus Rickettsia barbariae in ticks in Israel. Am J Trop Med Hyg 90: 920–922. 10.4269/ajtmh.13-0697 24615133 PMC4015588

[10] Roux V, Rydkina E, Eremeeva M, Raoult D (1997) Citrate synthase gene comparison, a new tool for phylogenetic analysis, and its application for the rickettsiae. Int J Syst Bacteriol 47: 252–261. 9103608 10.1099/00207713-47-2-252

[11] Roux V, Fournier PE, Raoult D (1996) Differentiation of spotted fever group rickettsiae by sequencing and analysis of restriction fragment length polymorphism of PCR-amplified DNA of the gene encoding the protein rOmpA. J Clin Microbiol 34: 2058–2065. 8862558 10.1128/jcm.34.9.2058-2065.1996 PMC229190

[12] Chmielewski T, Podsiadly E, Karbowiak G, Tylewska-Wierzbanowska S (2009) Rickettsia spp. in ticks, Poland. Emerg Infect Dis 15: 486–488. 10.3201/eid1503.080711 19239772 PMC2681112

[13] Roux V, Raoult D (1995) Phylogenetic analysis of the genus Rickettsia by 16S rDNA sequencing. Res Microbiol 146: 385–396. 8525055 10.1016/0923-2508(96)80284-1

[14] Feldman-Muhsam B (1951) A Note on East Mediterranean Species of the Haemaphysalis. Bull Res Counc Isr 1: 96–107.

[15] Feldman-Muhsam B (1954) Revision of the genus Hyalomma. Bull Res Counc Isr 64: 70–150.

[16] Pegram R, Clifford C, Walker J, Keirans J (1987) Clarification of the Rhipicephalus sanguineus Group (Acari, Ixodoidea, Ixodidae). I. R. sulcatus Neumann, 1908 and R. turanicus Pomerantsev, 1936. Syst Parasitol 10: 3–26.

[17] Apanaskevich DA, Schuster AL, Horak IG (2008) The genus Hyalomma: VII. Redescription of all parasitic stages of H. (Euhyalomma) dromedarii and H. (E.) schulzei (Acari: Ixodidae). J Med Entomol 45: 817–831. 18826023 10.1603/0022-2585(2008)45[817:tghvro]2.0.co;2

[18] Ogo NI, de Mera IG, Galindo RC, Okubanjo OO, Inuwa HM, et al (2012) Molecular identification of tick-borne pathogens in Nigerian ticks. Vet Parasitol 187: 572–577. 10.1016/j.vetpar.2012.01.029 22326937

[19] Paddock CD, Sumner JW, Comer JA, Zaki SR, Goldsmith CS, et al (2004) Rickettsia parkeri: a newly recognized cause of spotted fever rickettsiosis in the United States. Clin Infect Dis 38: 805–811. 14999622 10.1086/381894

[20] Anstead CA, Chilton NB (2013) A novel Rickettsia species detected in Vole Ticks (Ixodes angustus) from Western Canada. Appl Environ Microbiol 79: 7583–7589. 10.1128/AEM.02286-13 24077705 PMC3837795

[21] Beati L, Raoult D (1993) Rickettsia massiliae sp. nov., a new spotted fever group Rickettsia. Int J Syst Bacteriol 43: 839–840. 8240964 10.1099/00207713-43-4-839

[22] Babalis T, Tselentis Y, Roux V, Psaroulaki A, Raoult D (1994) Isolation and identification of a rickettsial strain related to Rickettsia massiliae in Greek ticks. Am J Trop Med Hyg 50: 365–372. 7908503 10.4269/ajtmh.1994.50.365

[23] Cardenosa N, Segura F, Raoult D (2003) Serosurvey among Mediterranean spotted fever patients of a new spotted fever group rickettsial strain (Bar29). Eur J Epidemiol 18: 351–356. 12803376 10.1023/a:1023654400796

[24] Mura A, Masala G, Tola S, Satta G, Fois F, et al (2008) First direct detection of rickettsial pathogens and a new rickettsia, 'Candidatus Rickettsia barbariae', in ticks from Sardinia, Italy. Clin Microbiol Infect 14: 1028–1033. 10.1111/j.1469-0691.2008.02082.x 19040474

[25] Sarih M, Socolovschi C, Boudebouch N, Hassar M, Raoult D, et al (2008) Spotted fever group rickettsiae in ticks, Morocco. Emerg Infect Dis 14: 1067–1073. 10.3201/eid1407.070096 18598627 PMC2600325

[26] Vitale G, Mansuelo S, Rolain JM, Raoult D (2006) Rickettsia massiliae human isolation. Emerg Infect Dis 12: 174–175. 16634183 10.3201/eid1201.050850 PMC3291392

[27] Parola P, Socolovschi C, Jeanjean L, Bitam I, Fournier PE, et al (2008) Warmer weather linked to tick attack and emergence of severe rickettsioses. PLoS Negl Trop Dis 2: e338 10.1371/journal.pntd.0000338 19015724 PMC2581602

[28] Garcia-Garcia JC, Portillo A, Nunez MJ, Santibanez S, Castro B, et al (2010) A patient from Argentina infected with Rickettsia massiliae. Am J Trop Med Hyg 82: 691–692. 10.4269/ajtmh.2010.09-0662 20348520 PMC2844561

[29] Tijsse-Klasen E, Hansford KM, Jahfari S, Phipps P, Sprong H, et al (2013) Spotted fever group rickettsiae in Dermacentor reticulatus and Haemaphysalis punctata ticks in the UK. Parasit Vectors 6: 212 10.1186/1756-3305-6-212 23870197 PMC3725166

[30] Abdel-Shafy S, Allam NA, Mediannikov O, Parola P, Raoult D (2012) Molecular detection of spotted fever group rickettsiae associated with ixodid ticks in Egypt. Vector Borne Zoonotic Dis 12: 346–359. 10.1089/vbz.2010.0241 22217182

[31] Palomar AM, Santibanez P, Mazuelas D, Roncero L, Santibanez S, et al (2012) Role of birds in dispersal of etiologic agents of tick-borne zoonoses, Spain, 2009. Emerg Infect Dis 18: 1188–1191. 10.3201/eid1807.111777 22709801 PMC3376802

[32] Keysary A, Eremeeva ME, Leitner M, Din AB, Wikswo ME, et al (2011) Spotted fever group rickettsiae in ticks collected from wild animals in Israel. Am J Trop Med Hyg 85: 919–923. 10.4269/ajtmh.2011.10-0623 22049050 PMC3205642

[33] Socolovschi C, Reynaud P, Kernif T, Raoult D, Parola P (2012) Rickettsiae of spotted fever group, Borrelia valaisiana, and Coxiella burnetii in ticks on passerine birds and mammals from the Camargue in the south of France. Ticks Tick Borne Dis 3: 355–360. 10.1016/j.ttbdis.2012.10.019 23141104

